# Assessing the diagnostic capability of the macular ganglion cell complex and retinal fiber layer thickness in glaucoma patients

**DOI:** 10.6026/973206300212570

**Published:** 2025-08-31

**Authors:** Tripti Thakur, Nishita Yadav, Nazia Imam, Ali M.S.

**Affiliations:** 1 Department of Regional Institute of Ophthalmology, Indira Gandhi Institute of Medical Sciences, Patna, Bihar, India

**Keywords:** Glaucoma, optical coherence tomography, ganglion cell complex, retinal nerve fiber layer, diagnostic accuracy, structural biomarkers

## Abstract

The diagnostic value of macular ganglion cell complex (GCC) and retinal nerve fibre layer (RNFL) thickness in glaucoma is of interest. Using OCT imaging, 90 subjects (45 glaucoma and 45 controls) were studied and the thickness of RNFL and GCC was evaluated. The RNFL was significantly lower in glaucoma patients (71.3 ± 6.5 µm) compared to controls (95.2 ± 5.9 µm, p< 0.001) and so was GCC thickness (72.1 ± 7.3 µm versus 90.6 ± 6.8 µm in controls, p< 0.001). The diagnostic accuracy of RNFL (AUC 0.89) was a bit higher than GCC (AUC 0.87). Combined evaluation enhanced the sensitivity of early glaucoma detection, as well as specificity.

## Background:

Glaucoma is a progressive and chronic ocular neuropathy characterised primarily by the irreversible loss of retinal ganglion cells (RGCs) and their optic nerve axons, resulting in vision field loss [1]. It is one of the leading causes of global blindness and its asymptomatic nature in the initial stages contributes to prolonged misdiagnosis [2]. Alterations in the optic nerve head (ONH), retinal nerve fibre layer (RNFL) and ganglion cell complex (GCC) manifest structurally prior to the detection of functional deficits using routine automated perimetry [3]. Optical coherence tomography (OCT) has transformed the management process of glaucoma early detection and monitoring because detailed cross-sectional images of retinal structures can be imaged in high resolution [4]. Peripapillary RNFL thickness is one of the numerous OCT parameters that are commonly used in the evaluation of glaucomatous loss. In recent years, there has been increased interest in studying macular GCC, which comprises the nerve fibre layer, ganglion cell layer and inner plexiform layer, as more than half of all RGCs are situated in the macular area [5]. In various studies, GCC thickness measurements have proved useful in early detection of glaucomatous changes when peripapillary RNFL does not appear abnormal [6, 7]. In addition, a combination of GCC and RNFL analysis could provide superior diagnostic functions, particularly to distinguish the presence of early glaucoma in an eye as compared to the healthy one [8]. Although the relative diagnostic value of GCC and RNFL has been growing, it is still the subject of research. Glaucoma-related axonal loss begins at the retinal ganglion cells, predominantly located in the macular region [5]. The anatomical integrity of these neurones is represented in the ganglion cell complex (GCC), comprising the retinal nerve fibre layer (RNFL), ganglion cell layer (GCL) and inner plexiform layer (IPL). Because the destruction of these macular layers is directly influenced by early glaucomatous harm, the thickness of GCC has been viewed as a potential structural biomarker [6]. Specifically, the sensitivity of macular ganglion cells to mild stages of disease explains why high-resolution imaging located in this area is required [7]. The SD-OCT enables accurate assessment of both the peripapillary RNFL and the macular GCC, so it is possible to monitor small structural losses that would not be shown on a fundus examination or visual field test [8]. Although RNFL thinning is a proven parameter of glaucoma progression, in some circumstances, when RNFL thinning has shown no above-normal values, even GCC measurement can reveal the damage earlier because of inter-individual anatomical variability [9]. Furthermore, GCC appears not to be influenced by disc dimensions or peripapillary atrophy as much as macular scans and thus, this parameter appears useful as an adjunct parameter in difficult-to-diagnose cases [10]. Past research has shown that there is a tight relation between GCC thinning and functional visual field loss in glaucomatous eyes [11]. Moreover, the combined analysis involving GCC and RNFL parameters has been found beneficial in the study compared to either specific parameter due to the enhancement in the accuracy of diagnosing the pre-perimetric glaucomatous condition [12]. Nevertheless, it has been introduced in a clinical setting, with GCC being underutilized in day-to-day clinical practice due to underdeveloped normative databases as well as insufficient sensitivity between different OCTs [13]. Therefore, additional evidence is required to support its ability to be used standalone as a diagnostic tool or even integrated into the clinical algorithms. Therefore, it is of interest to compare the diagnostic potential of macular GCC thickness with the peripapillary RNFL thickness of patients with primary open-angle glaucoma.

## Materials and Methods:

The research was a cross-sectional observational study conducted at the Department of Regional Institute of Ophthalmology, Indira Gandhi Institute of Medical Sciences, Patna, Bihar, India, involving diagnosed patients with primary open-angle glaucoma and age-matched healthy control subjects. A sample of 90 participants was utilised, comprising 45 patients with glaucoma and 45 individuals without the condition. The glaucoma cohort comprised individuals over 40 years diagnosed with primary open-angle glaucoma, characterised by open angles on gonioscopy, elevated intraocular pressure (>21 mmHg) and distinctive optic disc alterations (enhanced cup-to-disc ratio, neuroretinal rim thinning), accompanied by corresponding visual field deficits on standard automated perimetry (SAP). The control group exhibited normal intraocular pressure (IOP), a healthy optic nerve head and no history of ocular or systemic disorders that could potentially affect the retina or optic nerve. A comprehensive ophthalmic evaluation was conducted on all subjects, including best-corrected visual acuity (BCVA), slit-lamp biomicroscopy, applanation tonometry, gonioscopy, fundus examination and standard automated perimetry using the Humphrey Visual Field Analyser (24-2 SITA Standard). Only dependable visual field assessments were used, characterised by fixation losses of less than 20% and false positives and false negatives each below 20%. Optical coherence tomography imaging was conducted with a spectral-domain OCT device (Topcon). The optic disc scan was collected using the 6 x 6 mm protocol at a resolution of 512 x 256, while the macular scan was taken using the 7 x 7 mm cube technique at the same resolution of 512 x 256. The peripapillary retinal nerve fibre layer (RNFL) thickness was evaluated via the disc scan, while the macular ganglion cell complex (GCC) was analysed utilising the macular scan. Scans with a signal strength below 7, artefacts, or segmentation inaccuracies were omitted. The average, superior and inferior retinal nerve fibre layer (RNFL) and ganglion cell complex (GCC) thicknesses were documented and examined. All statistical analyses were conducted using SPSS software (version 25.0). Descriptive statistics for continuous variables were presented as means and standard deviations (SD) and independent t-tests were employed to compare the groups. The diagnostic efficacy was assessed using receiver operating characteristic (ROC) curves and the area under the curve (AUC) was computed. A P-value of less than 0.05 was employed as the criterion for statistical significance.

## Results:

A total of 90 participants were enrolled in the study, including 45 patients with primary open-angle glaucoma and 45 age-matched healthy controls. The mean age of glaucoma patients was 58.3 ± 7.6 years, while that of the control group was 56.9 ± 6.9 years, with no statistically significant difference (p = 0.28). Both groups were comparable in terms of gender distribution and refractive status. Table 1 (see PDF) shows the comparison of mean retinal nerve fiber layer (RNFL) thickness parameters between the glaucoma and control groups. Glaucoma patients exhibited significantly reduced average RNFL thickness (71.3 ± 6.5 µm) compared to controls (95.2 ± 5.9 µm, p< 0.001). Similarly, superior and inferior quadrant RNFL values were also markedly lower in the glaucoma group (p< 0.001). The macular ganglion cell complex (GCC) thickness was also significantly lower in the glaucoma group when compared with the control group, as illustrated in Table 2 (see PDF). The average GCC thickness in glaucoma eyes was 72.1 ± 7.3 µm versus 90.6 ± 6.8 µm in healthy eyes (p< 0.001). Both superior and inferior GCC segments showed statistically significant thinning in glaucomatous eyes. To assess the diagnostic accuracy of RNFL and GCC parameters, receiver operating characteristic (ROC) curve analysis was performed. As shown in Table 3 (see PDF), the area under the curve (AUC) for average RNFL thickness was 0.89, indicating high diagnostic ability. GCC parameters also demonstrated good diagnostic performance, with an AUC of 0.87. Combining both RNFL and GCC values slightly improved overall sensitivity and specificity. These findings demonstrate that both RNFL and GCC thickness measurements are effective in detecting glaucomatous changes, with RNFL offering slightly higher diagnostic accuracy (Tables 1-3 - see PDF).

## Discussion:

This research aimed to ascertain and contrast the diagnostic efficacy of macular ganglion cell complex (GCC) thickness and peripapillary retinal nerve fibre layer (RNFL) thickness in individuals with primary open-angle glaucoma, utilising spectral-domain optical coherence tomography (SD-OCT). We exhibited a significant disparity in RNFL and GCC thickness between glaucomatous eyes and normal controls, supporting the idea that structural loss in glaucoma is accurately assessed by OCT. The average RNFL thickness revealed a significant reduction in the glaucomatous patients compared to controls, as already mentioned in previous studies where RNFL thinning was cited as one of the characteristics of glaucomatous optic neuropathy [1, 2]. The RNFL thinning is the reflection of axonal degeneration correlated with the field loss and disease progression [3]. The average RNFL thickness proved to be quite diagnostic in this study, with an area under the curve of 0.89, making it clear that it is one of the first structural signs as far as the diagnosis of glaucoma is concerned [4]. In the glaucoma group, macular GCC thickness was also significantly low. The intensity of retinal ganglion cells in the macular area is high and that is the reason why GCC analysis has acquired significance in early detection of glaucoma [5, 6]. It has also been emphasized that the RNFL damage may be detectable after macular GCC thinning has already taken place, particularly in pre-perimetric glaucoma or with cases of minimal disorders on the disc [7, 8]. The difference between the AUCs of GCC thickness in our study and the studies by Mwanza *et al.* and Kim *et al.* indicates that GCC thickness has a good diagnostic value in the diagnosis of asthma [9, 10]. Despite RNFL yielding a slightly higher level of sensitivity and specificity than GCC, adding both parameters together demonstrated an even better diagnostic performance (AUC 0.91), which is consistent with the previous findings that multiparametric analysis is commercially used to ensure a superior identification of glaucoma [11, 12]. The integrated solution can circumnavigate some of these problems because of their anatomical variations displayed in the shape of the optic disc or signal strength problems during isolated measurements [13]. Furthermore, it has the advantage of boosting the faith in the diagnosis of borderline or early cases of glaucoma, where deviations regarding the single parameters can be slight [14, 15]. The weaknesses of the study are that it is cross-sectional and uses a comparatively small number of individuals in the sample. Evaluation of the trend of GCC and RNFL decrease with time would require longitudinal studies. Secondarily, Differences in reproducibility across clinical environments may be attributable to OCT device algorithms and algorithms computing segmentation software. Still, the results support the usefulness of SD-OCT when it comes to offering an objective and repeatable measure of retinal structures in the setting of glaucoma diagnosis and tracking.

## Conclusion:

Both the macular and peripapillary measurements in GCC and RNFL thickness are helpful to diagnose glaucoma. Although RNFL remains the gold standard measurement of the structural assessment, the GCC analysis provides supplemental data, especially during the initial stages of the development of the disease. With the inclusion of the two parameters into medical practice, the early diagnosis of glaucoma could be enhanced, effective, timely intervention could be achieved and finally, visual functions could be preserved among glaucoma patients.
